# The role of inflammation in schizophrenia

**DOI:** 10.3389/fnins.2015.00372

**Published:** 2015-10-21

**Authors:** Norbert Müller, Elif Weidinger, Bianka Leitner, Markus J. Schwarz

**Affiliations:** ^1^Department of Psychiatry and Psychotherapy, Ludwig Maximilian UniversityMunich, Germany; ^2^Department of Laboratory Medicine, Ludwig Maximilian UniversityMunich, Germany

**Keywords:** inflammation, schizophrenia, psychoneuroimmunology, neuroinflammation, COX-2

## Abstract

High levels of pro-inflammatory substances such as cytokines have been described in the blood and cerebrospinal fluid of schizophrenia patients. Animal models of schizophrenia show that under certain conditions an immune disturbance during early life, such as an infection-triggered immune activation, might trigger lifelong increased immune reactivity. A large epidemiological study clearly demonstrated that severe infections and autoimmune disorders are risk factors for schizophrenia. Genetic studies have shown a strong signal for schizophrenia on chromosome 6p22.1, in a region related to the human leucocyte antigen (HLA) system and other immune functions. Another line of evidence demonstrates that chronic (dis)stress is associated with immune activation. The vulnerability-stress-inflammation model of schizophrenia includes the contribution of stress on the basis of increased genetic vulnerability for the pathogenesis of schizophrenia, because stress may increase pro-inflammatory cytokines and even contribute to a lasting pro-inflammatory state. Immune alterations influence the dopaminergic, serotonergic, noradrenergic, and glutamatergic neurotransmission. The activated immune system in turn activates the enzyme indoleamine 2,3-dioxygenase (IDO) of the tryptophan/kynurenine metabolism which influences the serotonergic and glutamatergic neurotransmission via neuroactive metabolites such as kynurenic acid. The described loss of central nervous system volume and the activation of microglia, both of which have been clearly demonstrated in neuroimaging studies of schizophrenia patients, match the assumption of a (low level) inflammatory neurotoxic process. Further support for the inflammatory hypothesis comes from the therapeutic benefit of anti-inflammatory medication. Metaanalyses have shown an advantageous effect of cyclo-oxygenase-2 inhibitors in early stages of schizophrenia. Moreover, intrinsic anti-inflammatory, and immunomodulatory effects of antipsychotic drugs are known since a long time. Anti-inflammatory effects of antipsychotics, therapeutic effects of anti-inflammtory compounds, genetic, biochemical, and immunological findings point to a major role of inflammation in schizophrenia.

## Introduction

We humans are constantly being assaulted by infectious agents, noxious chemicals, and physical traumata. Fortunately, we have evolved a complex process, the inflammatory response, to help fight and clear infection, remove damaging chemicals, and repair damaged tissue (O'Neill, 2008). The harmful effects of inflammation can be observed in many infectious and autoimmune diseases. The interactions between environmental factors and genetically encoded components of the inflammatory response determine whether the outcome will be health or disease.

As in other sites of the body, inflammation in the central nervous system (CNS) has a dual role, i.e., it may be neuroprotective or neurotoxic (Hohlfeld et al., 2007). While acute inflammation in the CNS (e.g., acute encephalitis) leads to life-threatening states within hours or days, chronic inflammation might be associated with impairment over months, years, or a lifetime.

As an example, multiple sclerosis (MS) is an inflammatory disease of the CNS that shows a relapsing-remitting course and, in a certain percentage of patients, also a chronic, progressive course. Parallels between MS and schizophrenia, which also often shows a chronic course, have repeatedly been highlighted as arguments for similar pathogenetic mechanisms in these disorders (Hanson and Gottesman, 2005).

The concept of “smoldering inflammation” implies that CNS inflammation drives the disease process in both acute and chronic stages (Kutzelnigg et al., 2005). While during acute inflammation the peripheral immune system interacts closely with the CNS, which is invaded by macrophages and B and T cells, in chronic processes the immune response in the CNS is thought to be increasingly secluded from the peripheral immune system (“compartmentalization” of the inflammatory process; Meinl et al., 2008; Kerschensteiner et al., 2009). Chronic MS, for example, is primarily characterized by disseminated activation of microglial cells.

There are numerous descriptions of an association between infection, chronic inflammation of the CNS, and schizophrenia (Anderson et al., 2013). For example, symptoms of schizophrenia have been described in the encephalitic form of MS (Felgenhauer, 1990), in viral CNS infection with herpes simplex virus type 1 (HSV-1; Chiveri et al., 2003), HSV-2 (Oommen et al., 1982), and measles (Hiroshi et al., 2003) and also in autoimmune processes such as poststreptococcal disorders (Mercadante et al., 2000; Bechter et al., 2007; Kerbeshian et al., 2007; Teixeira et al., 2007), lupus erythematodes, and scleroderma (van Dam, 1991; Müller et al., 1992, 1993; Nikolich-Zugich, 2008).

## Inflammatory mechanisms in the CNS

Inflammation in the CNS is mediated by pro-inflammatory cytokines, microglial cells (resident macrophages in the brain), astrocytes, and invading immune cells such as monocytes, macrophages, and T or B lymphocytes. Although a well-regulated inflammatory process is essential for tissue homeostasis and proper function, an excessive inflammatory response can be the source of additional injury to host cells. Uncontrolled inflammation may be the result of either infectious agents (e.g., bacteria, viruses) or a reaction to neuronal lesions from traumata, a genetic defect or environmental toxins.

## Microglia as an important cellular basis of inflammation in the CNS

Microglia comprise ~15% of the total CNS cells and are the primary component of the intrinsic immune system in the CNS, where they provide the first line of defense after injury or disease and are the principal component of neuroinflammation.

Microglia can be activated in different ways:

1. A systemic inflammatory challenge triggers microglia activation, resulting in the release of proinflammatory cytokines in the CNS, which can mediate “sickness behavior” (Dantzer, 2001) and other mental states. Microglia play a role in the synthesis of these central cytokines (van Dam, 1991).
2. Microglia are “sensitized” or “primed” (Perry, 2007) by different stimuli, including neurodegeneration (Cunningham et al., 2005), aging (Godbout and Johnson, 2009), and stress (Sparkman and Johnson, 2008). This process of sensitization or priming results in the elicitation of an exaggerated immune response to a weak stimulus. After priming, a second stimulus, e.g., low systemic inflammation or stress, may lead to microglia proliferation and increased production of proinflammatory cytokines by microglia (Frank et al., 2007). This exaggerated cytokine response may result in acute changes in behavior by exacerbating or re-exacerbating an inflammatory pathology in the CNS.

## Kindling and sensitization of the immune response: the basis for the stress-induced inflammatory response in psychiatric disorders

The immune response and the release of cytokines can become more sensitized to activating stimuli by a kindling process: the initial immune response, i.e., the release of cytokines and other mediators of immune activation, is initiated as a result of exposure to a certain stimulus; thereafter, either re-exposure to the same stimulus, e.g., stress or infection, is associated with an increased release of cytokines or a weaker stimulus is necessary for the same activation process. This “sensitization” or “kindling” may be due to the memory function of the acquired immune system (Furukawa et al., 1998; Sparkman and Johnson, 2008). Stress-associated release of IL-6 was shown to reactivate (prenatally) conditioned processes (Zhou et al., 1993). In healthy persons, a second stimulus (e.g., systemic inflammation, stress) led to immune activation, associated with cellular proliferation, and increased production and release of proinflammatory cytokines (Frank et al., 2007). This mechanism is a key mechanism for triggering an immune activation and inflammation, e.g., the stress-induced immune activation leading to psychopathological symptoms. A sensitization process in the immune system is in accordance with the view that after an infection during early childhood, re-infection or another stimulation of the immune system in later stages of life might be associated with a boosted release of sensitized cytokines, resulting in neurotransmitter disturbances.

Sensitization phenomena play a role in stress-related, cytokine-induced, neurotransmitter-mediated behavioral abnormalities, i.e., the cytokine response to a stimulus increases while the intensity of the stimulus decreases (Sparkman and Johnson, 2008). In animal experiments, however, cytokines promote greater neurotransmitter responses when the animals are re-exposed to the cytokine (Anisman and Merali, 2003), for example TNF-α (Hayley et al., 2002). In the CNS, the stress-induced activation and proliferation of microglia may mediate these cytokine effects (Nair and Bonneau, 2006).

## The vulnerability-stress-inflammation model of schizophrenia

The vulnerability-stress model of psychiatric disorders, first postulated for schizophrenia more than 30 years ago (Zubin and Spring, 1977), focuses on the role of physical and mental stress in triggering a psychotic episode. This model says that in especially vulnerable individuals (e.g., genetically vulnerable) stress represents an additional risk for the outbreak of the schizophrenic disease. An increased vulnerability of the offspring was shown—in addition to genetic vulnerability—if an inflammatory response was induced in the mother during the second trimester of pregnancy or in the offspring during later stages of CNS development in animal models (Giovanoli et al., 2013; Green et al., 2014). A recent study, the first large-scale epidemiological study in psychiatry, showed, however, that severe infections and autoimmune disorders increase additively the risk of schizophrenia and schizophrenia spectrum disorders also in adults (Benros et al., 2011).

The underlying mechanisms of the co-occurrence of stress and inflammation were studied in animal experiments and stress was repeatedly shown to be associated with an increase in pro-inflammatory cytokines (Sparkman and Johnson, 2008).

The specific influence of the inflammatory mechanisms on neurotransmitter systems in schizophrenia will be discussed below. Moreover, the modulation of glutamatergic neurotransmission will be highlighted; glutamate is the most abundant neurotransmitter in the CNS and differ-entially involved via cytokine-directed tryptophan/kynurenine metabolism in schizophrenia, presumably but not exclusively mediated by NMDA receptors. Besides others, genetic factors of the kynurenine metabolites play a role (Claes et al., 2011).

## The immune dysbalance in schizophrenia is associated with chronic inflammation

Degradation products of inflammatory substances have been described in schizophrenic brain tissue (Körschenhausen et al., 1996) and in the CSF of about 50% of schizophrenia patients (Wildenauer et al., 1991).

Regarding the cytokine pattern in schizophrenia, a blunted type 1 [e.g., Interferon(IFN)-gamma, Interleukin(IL)-2, soluble IL-2 receptors] and (compensatory) increased type 2 cytokine pattern (e.g., IL-6, IL-10) have been repeatedly observed in unmedicated schizophrenia patients (Müller and Schwarz, 2006). These findings point to an imbalance of the type 1 and type 2 immune responses in schizophrenia. Overviews on the imbalance in schizophrenia of the type 1 and type 2 and the pro- and anti-inflammatory immune systems as well as innate immunity, including the monocyte/macrophage system (Sperner-Unterweger et al., 1992), have recently been published and indicate that an inflammatory process plays an important role in the pathophysiology of at least a subgroup of schizophrenia patients (Potvin et al., 2008; Müller and Schwarz, 2010; Miller et al., 2011). Accordingly, first pilot experiences with the type 1-stimulating substance interferon-gamma (IFN-γ) as a therapeutic approach in schizophrenia are encouraging (Grüber et al., 2014).

## The impact of inflammation on neurotransmitters in schizophrenia

Over the last five decades, research on the neurobiology of schizophrenia has focused overwhelmingly on disturbances of dopaminergic neurotransmission (Carlsson, 1988). There is no doubt that a dysfunction of the dopamine system is involved in the pathogenesis of schizophrenia, although the mechanism is not clear and antidopaminergic antipsychotic drugs still show unsatisfactory therapeutic effects.

IL-1β, which can induce the conversion of rat mesencephalic progenitor cells into a dopaminergic phenotype (Kabiersch et al., 1998; Ling et al., 1998; Potter et al., 1999), and IL-6, which is highly effective in decreasing the survival of fetal brain serotonergic neurons (Jarskog et al., 1997), seem to have an important influence on the development of the neurotransmitter systems involved in schizophrenia, although the specificity of these cytokines is a matter of discussion. Maternal immune stimulation during pregnancy was shown to increase the number of mesencephalic dopaminergic neurons in the fetal brain (Winter et al., 2009); the increase was probably associated with a dopaminergic excess in the midbrain (Winter et al., 2009). Persistent pathogens might be key factors that drive imbalances of the immune reaction (Nikolich-Zugich, 2008). Nevertheless, many questions about how immunity and immune pathology are involved in virus infections remain unanswered (Rouse and Sehrawat, 2010).

Much evidence seems to indicate that a lack of glutamatergic neurotransmission, mediated via NMDA antagonism, is a key mechanism in the pathophysiology of schizophrenia (Müller and Schwarz, 2007; Genius et al., 2013; Goff, 2015; Howes et al., 2015). The only NMDA receptor antagonist known to occur naturally in the human CNS is kynurenic acid (Stone, 1993), one of at least three neuroactive intermediate products of the kynurenine pathway. A predominant type 2 immune response inhibits the enzyme indoleamine 2,3-dioxygenase (IDO), resulting in an increased production of kynurenic acid in schizophrenia and in NMDA receptor antagonism (Müller and Schwarz, 2007; Müller et al., 2011). The recent finding of NMDA receptor antibodies in about 10% of acute (unmedicated) schizophrenia patients is especially interesting in this regard (Vincent and Bien, 2008; Steiner et al., 2013).

Discrepancies in the findings regarding kynurenic acid in schizophrenia, however, have to be discussed (Kegel et al., 2014). Elevated kynurenic acid has mainly been described in the CSF (Erhardt et al., 2001; Linderholm et al., 2012), in the brains of schizophrenia patients (Schwarcz et al., 2001; Sathyasaikumar et al., 2011) and in animal models of schizophrenia (Olsson et al., 2009). However, no increased kynurenic acid levels were observed in the peripheral blood of first-episode schizophrenia patients (Condray et al., 2011) and other groups of schizophrenia patients (Myint et al., 2011). In a toxoplasma animal model the relationship between IDO, infection, kynurenine metabolites, and schizophrenia is exemplified (Notarangelo et al., 2014). Antipsychotic medication, however, influences kynurenine metabolites and has to be regarded as an interfering variable (Ceresoli-Borroni et al., 2006; Condray et al., 2011; Myint et al., 2011).

## Infection as a risk factor schizophrenia

Prenatal immune activation—infection triggered or not—is an important risk factor for schizophrenia (Meyer et al., 2011). Animal models of schizophrenia show that stimulation of the maternal immune system during pregnancy by viral or bacterial agents leads to typical (schizophrenia-like) symptoms, i.e., the disturbed prepulse inhibition in the offspring (Meyer and Feldon, 2009; Meyer et al., 2011).

Evidence for pre- or perinatal exposure to infections as a risk factor for schizophrenia has not only been obtained from animal models (Westergaard et al., 1999; Buka et al., 2001). Also in humans studies of infections as risk factors for schizophrenia have been performed on several viral disorders (Pearce, 2001; Brown et al., 2004a; Buka et al., 2008). An increased risk for schizophrenia in the offspring was observed after respiratory infections (Brown et al., 2000; Sørensen et al., 2009), genital infections, and reproductive tract infections (Babulas et al., 2006; Sørensen et al., 2009). Specifically, women during pregnancy infected with *Toxoplasma gondii* were intensely studied as risk factor for schizophrenia (Brown et al., 2005).

Infections before birth increase the risk for later schizophrenia (Gattaz et al., 2004; Boksa, 2008; Brown, 2008; Dalman et al., 2008), as do infections—in particular CNS infections—during later stages of brain development. Antibody titers against viruses have been examined in the sera of schizophrenia patients for many years (Yolken and Torrey, 1995). The results, however, have been inconsistent, e.g., because interfering factors were not controlled for. Antibody levels are associated with the medication state, a finding which partly explains the earlier controversial results (Leweke et al., 2004). In one of our own studies, higher titers of different pathogens were found in schizophrenia patients than in controls, a phenomenon that we called “infectious index” (Krause et al., 2010).

In humans, increased maternal levels of the proinflammatory cytokine interleukin-8 (IL-8) during pregnancy were shown to be associated with an increased risk for schizophrenia in the offspring, whatever the reason for the increase in IL-8 (Brown et al., 2004b). Moreover, increased maternal IL-8 levels in pregnancy were also significantly related to decreased brain volume, i.e., lower volumes of the right posterior cingulum and left entorhinal cortex and higher volumes of the ventricles in the schizophrenic offspring (Ellman et al., 2010).

A recent study, the first large-scale epidemiological study in psychiatry, showed, however, that severe infections and autoimmune disorders increase additively the risk of schizophrenia and schizophrenia spectrum disorders (Benros et al., 2011). This is an important finding, since mostly maternal infections during pregnancy had been studied before (in animal models). Infections after birth or during childhood and adolescence in later schizophrenic diagnosed patients—i.e., lifetime infections of the schizophrenia patients—have only rarely been studied (Benros et al., 2011, 2012). The sensitivity of the study in recording infections was not high, because only infections leading to hospital admissions were recorded. Normally, only extraordinary severe infections lead to a hospital contact. Therefore, despite the large scale of the study, it may have clearly identified only the “tip of an iceberg” of risk factors (Benros et al., 2012).

## CNS volume loss in imaging studies—a consequence of an inflammatory process?

Gross inflammatory changes have not been found in neuroimaging or neuropathological studies of schizophrenia. However, there is no doubt that a decreased CNS volume can be observed as early as the first episode and a progressive loss in CNS volume occurs during the further course of the disease (Chakos et al., 2005; Job et al., 2006; Steen et al., 2006; Gogtay et al., 2008). Moreover, a relationship was described between volume loss and an increased genetic risk for a higher production of the immune marker IL-1β (Meisenzahl et al., 2001); the relationship between maternal IL-8 levels and CNS volume was mentioned above (Ellman et al., 2010).

The ligand PK 11195 is used in positron emission tomography (PET) to estimate microglial activation (Versijpt et al., 2003). In schizophrenia, an increased expression of PK 11195 was shown to be a marker of an inflammatory process in the CNS (van Berckel et al., 2008; Doorduin et al., 2009). Moreover, positive correlations were also observed between expression of the microglial activation marker DAA1106 and both schizophrenia positive symptoms and duration of the disease (Takano et al., 2010).

## Cyclooxygenase-2 (COX-2) inhibition as an anti-inflammatory therapeutic approach in schizophrenia

Modern anti-inflammatory agents have been explored in schizophrenia. The cyclooxygenase-2 (COX-2) inhibitor celecoxib was studied in a prospective, randomized, double-blind study of acute exacerbations of schizophrenia. The patients receiving celecoxib add-on to risperidone showed a statistically significantly better outcome than the patients receiving risperidone alone; the clinical effects of COX-2 inhibition in schizophrenia were especially pronounced in cognition (Müller et al., 2005). The efficacy of therapy with a COX-2 inhibitor seems most pronounced in the first years of the schizophrenic disease process (Müller, 2010; Müller et al., 2010). A recent study also demonstrated a beneficial effect of acetylsalicylic acid in schizophrenic spectrum disorders (Laan et al., 2010). A meta-analysis of the clinical effects of non-steroidal anti-inflammatory drugs in schizophrenia revealed significant effects on schizophrenic total, positive and negative symptoms (Sommer et al., 2012), while another meta-analysis found a significant benefit only in schizophrenia patients with a short duration of disease or in first manifestation schizophrenia (Nitta et al., 2013).

## Further immune-related substances in the therapy of schizophrenia

Because of the role of microglia activation in inflammation, minocycline, an antibiotic and inhibitor of microglia activation, is an interesting substance for the treatment of schizophrenia. The improvement of cognition by minocycline has been described in animal models of schizophrenia (Mizoguchi et al., 2008) and in two double-blind, placebo-controlled add-on therapy trials in schizophrenia patients (Levkovitz et al., 2010; Chaudhry et al., 2012). In clinical studies, positive effects on schizophrenic negative symptoms were noted as well (Chaudhry et al., 2012). Case reports documented positive effects of minocycline on the whole symptom spectrum in schizophrenia (Ahuja and Carroll, 2007).

Acetylcysteine (ACC) and other substances, including omega-3 fatty acids, that have anti-inflammatory and other effects also provide some benefit to schizophrenia patients (overview: Sommer et al., 2014)

First pilot experiences with cytokine interferon gamma (IFN-γ), which stimulates the monocytic type 1 immune response, as a therapeutic approach in schizophrenia are encouraging (Grüber et al., 2014), although side effects, including unwanted immune effects, have to be carefully monitored and the results are only preliminary. On the other hand, such a hypothesis-driven therapeutic approach opens interesting perspectives for the development of therapeutic substances based on etiopathology.

## Methodological aspects of the response to immune-based therapy in schizophrenia

The knowledge that schizophrenia is a syndrome and that different pathological mechanisms may play a role in the disorder seems to indicate that immune pathology is restricted to a subgroup of patients. Although several biological markers, including immune markers, are discussed to reflect subgroups of schizophrenia, so far no marker is established for an immune-related schizophrenia. Accordingly, an immune-based therapeutic approach might be effective only in a subgroup of patients or, put another way, an immune-based therapy can be expected to show only a small therapeutic effect in an unselected group of schizophrenia patients. Another relevant point is that all clinical studies with immune-based treatment are add-on studies to an established standard therapy with antipsychotics. For ethical reasons this design has to be used until the add-on substance has a proven effect in schizophrenia. In order to show superiority over an effective antipsychotic in monotherapy, however, the add-on substance has to have a huge additional effect before it reaches statistical significance over placebo and the antipsychotic. In the light of high and increasing placebo response rates in schizophrenia studies (Rutherford et al., 2014), anti-inflammatory substances need high effect sizes to show statistical superiority in double-blind, randomized, placebo-controlled studies. Moreover, schizophrenia patients participating in clinical studies, and especially in studies with a new, unproven therapeutic approach, often show an unfavorable, sometimes therapy-resistant course of the disorder, i.e., several of the studies may include a “negative” selection of severely ill patients.

The above mentioned methodological aspects may explain the difficulties in showing a convincing effect of anti-inflammatory drugs in schizophrenia.

## Conclusion

The possible influence on the pathogenesis of schizophrenia of an immunological process resulting in inflammation has long been neglected. Increasing evidence for a role of proinflammatory cytokines in schizophrenia, the strong influence of pro- and anti-inflammatory cytokines on tryptophan/kynurenine metabolism, the related influence of cytokines on glutamatergic neurotransmission, the results of imaging studies, genetic findings and, last but not least, the therapeutic effect of anti-inflammatory drugs all support the view that the recent increased focus of schizophrenia research on psychoneuroimmunology and inflammation is justified. On the other hand, one has to consider that immunological research is susceptible to artifacts, i.e., interfering variables such as medication, smoking, stress, sleep, and others play an important role and cannot always be controlled. This is exemplified by stress, which, according to the “vulnerability-stress model,” is not only a condition sine qua non in schizophrenia but is also a confounding factor in research of the immune system and inflammatory processes. The situation is similar for neuro-imaging studies: volume loss might be the result of different pathological processes other than inflammatory ones. Nevertheless, the results of these studies are encouraging and further studies should focus on the relationship between inflammatory markers in the blood and CSF and volume loss in the CNS. Moreover, the influence of different disease stages in schizophrenia might also have been neglected. The syndrome of schizophrenia is discussed to have different underlying pathological processes. Inflammation, however, also includes different stages and processes ranging from acute to chronic inflammation, including an autoimmune process.

These considerations show that although a lot of further research is necessary to clarify the role of the immune system in schizophrenia, recent findings encourage continued emphasis on this fascinating field.

## Funding

This work was supported by the Foundation “Immunität und Seele”.

### Conflict of interest statement

The authors declare that the research was conducted in the absence of any commercial or financial relationships that could be construed as a potential conflict of interest.
